# Mutations in the *miR-142* gene are not common in myeloproliferative neoplasms

**DOI:** 10.1038/s41598-022-15162-1

**Published:** 2022-06-28

**Authors:** Paulina Galka-Marciniak, Zuzanna Kanduła, Adrian Tire, Wladyslaw Wegorek, Kinga Gwozdz-Bak, Luiza Handschuh, Maciej Giefing, Krzysztof Lewandowski, Piotr Kozlowski

**Affiliations:** 1 Institute of Bioorganic Chemistry, Polish Academy of Sciences, Poznan, Poland; 2 Department of Hematology and Bone Marrow Transplantation, Poznan University of Medical Sciences, Poznan, Poland; 3 Institute of Computing Science, Poznan University of Technology, Poznan, Poland; 4 Institute of Human Genetics, Polish Academy of Sciences, Poznan, Poland

**Keywords:** Cancer genetics, Haematological cancer, Mutation, RNAi

## Abstract

Recent data indicate that *MIR142* is the most frequently mutated miRNA gene and one of the most frequently mutated noncoding elements in all cancers, with mutations occurring predominantly in blood cancers, especially diffuse large B-cell lymphoma (DLBCL) and follicular lymphoma. Functional analyses show that the *MIR142* alterations have profound consequences for lympho- and myelopoiesis. Furthermore, one of the targets downregulated by miR-142-5p is *CD274*, which encodes PD-L1 that is elevated in many cancer types, including myeloproliferative neoplasms (MPNs). To extend knowledge about the occurrence of *MIR142* mutations, we sequenced the gene in a large panel of MPNs [~ 700 samples, including polycythemia vera, essential thrombocythemia, primary myelofibrosis (PMF), and chronic myeloid leukemia], neoplasm types in which such mutations have never been tested, and in panels of acute myeloid leukemia (AML), and chronic lymphocytic leukemia (CLL). We identified 3 mutations (one in a PMF sample and two others in one CLL sample), indicating that *MIR142* mutations are rare in MPNs. In summary, mutations in *MIR142* are rare in MPNs; however, in specific subtypes, such as PMF, their frequency may be comparable to that observed in CLL or AML.

## Introduction

The miR-142 gene (*MIR142*) along with the well-known *TERT* promoter has recently been recognized as the most convincingly documented noncoding element consistently mutated in cancer^1,2^. However, unlike the *TERT* promoter, which is commonly mutated in almost all human malignancies^3^, *MIR142* is mutated almost exclusively (specifically) in lymphoid and myeloid malignancies, including chronic lymphocytic leukemia (CLL; 1–4%), follicular lymphoma (FL; 14–25%), diffuse large B-cell lymphoma (DLBCL; 20–27%), and acute myeloid leukemia (AML; 0.5–2% of cases) (^4^ and references therein).

Our recent cumulative study showed that although these mutations are distributed over the entire *MIR142* gene (i.e., the sequence coding for the stem-loop structure of the pre-miR-142 precursor, including flanks), they mostly cluster around the miR-142-3p seed^4^ and are strongly enriched in hematologic malignancies, which suggests that the mutations are functionally deleterious. Functional analysis of specific mutations (n.55A > G and n.58G > C) located in the miR-142-3p seed revealed that the mutations affect the miRNAs generated from both arms, resulting in loss of the ability of miR-142-3p to recognize/downregulate its targets and in lowering the level of miR-142-5p. More specifically, it was shown that by releasing repression of *ASH1L* (target of miR-142-3p), *MIR142* mutations cause sustained *HOXA9*/*A10* expression and alteration in the differentiation of hematopoietic progenitors (myeloid cell lines expansion, impaired erythropoiesis, and T cell lymphopenia), ultimately contributing to leukemic transformation^5^. It was also shown in the mouse model that mutations in *MIR142* synergize with mutations in *Idh2* to initiate AML^5,6^. It may be expected that deleterious *MIR142* mutations also affect other well-documented hematologic targets of miR-142-3p or miR-142-5p, including *RAC1* (Rac Family Small GTPase 1)^7^, *PROM1* encoding CD133 antigen^8,9^, *TNFRSF13C* encoding B cell-activating factor receptor (BAFF-R)^10^, *SOCS1* (Suppressor of Cytokine Signaling 1)^11,12^, *PTEN* (Phosphatase And Tensin Homolog)^13,14^, *IL6* (Interleukin 6)^15^ and *CD274* encoding programmed death-ligand 1 (PD-L1)^14,16,17^ which is an important immune checkpoint molecule that is elevated in many cancers, including different types of MPNs^18,19^. In light of results to date, miR-142 has emerged as an antitumor immunity-regulating factor important in different types of blood cancers, mostly lymphoid neoplasms.

In this study, to extend and complement knowledge about the occurrence and/or frequency of *MIR142* mutations in hematologic cancers, we sequenced *MIR142* in large panels of MPNs [including polycythemia vera (PV), essential thrombocythemia (ET), primary myelofibrosis (PMF), and chronic myeloid leukemia (CML)], blood disorders for which such mutations have never been tested, and in AML and CLL, for which the mutations have been reported.

## Methods

### Cancer sample collection

We analyzed DNA from 929 confirmed blood cancer samples diagnosed at the Department of Hematology and Bone Marrow Transplantation, Poznan University of Medical Sciences, Poland (collected in 2018–2021). The DNA was extracted from the patient’s peripheral blood cells using Syngen Blood/Cell Mini kit (Syngen Biotech, Poland). The study was approved by the Bioethics Committee of the Poznan University of Medical Sciences, Poland (No. 1037/09, 1056/16, 181/18, 846/21) and was conducted in accordance with the Declaration of Helsinki. Informed consent was obtained from all patients enrolled in this study. The samples were anonymized and then analyzed.

### Mutation nomenclature and target prediction

All mutations were designated (i) according to the Human Genome Variation Society (HGVS) nomenclature in relation to miRNA precursors, as deposited in miRbase release 22.1 and (ii) according to genomic position (hg38). Note that as *MIR142* is encoded by the minus strand, the nucleotide numbering in HGVS and genomic annotations is in the opposite directions; additionally, substitution designations are complementary to each other, e.g., A>G in the genome annotation is T>C in the transcript-based (HGVS) annotation. miRNA target predictions were performed with the TargetScan Custom (release 5.2) online tool.

### miRNA gene amplification and Sanger sequencing

The miR-142 gene was amplified by PCR using the following primers, miR142_F: 5'CTCACCTGTCACACGAGGTC3’, miR142_R: 5'CTCTTGGAGCAGGAGTCAGG3’ (231 bp product, annealing temperature 60 °C), enabling sequencing of the entire pre-miR-142 sequence together with ± 25 nt flanking regions. PCR was performed according to the manufacturer’s recommendations (GoTaq G2 Hot Start DNA Polymerase protocol, Promega, Madison, WI, USA). PCR products were purified using the EPPIC Fast kit (A&A Biotechnology, Gdynia, Poland) and sequenced directly using the BigDye v3.1 kit (Applied Biosystems, Foster City, CA, USA) with an ABI PRISM 3130xl genetic analyzer (Applied Biosystems, Foster City, CA, USA). The sequences were analyzed manually and with the use of Mutation Surveyor software (SoftGenetics, State College, PA, USA). All detected mutations were confirmed by sequencing in two directions.

### Additional molecular analyses

The analysis of the *JAK2*V617F mutation and *BCR-ABL* transcript was performed using real-time quantitative allele-specific RQ-PCR, and multiplex RT-PCR, respectively, as described before^20,21^. The mutations in *CALR* exon 9, *MPL* exon 10, *SRSF2* exon 1, *U2AF1* exons 2 and 6, *IDH1* exon 4, *IDH2* exon 4, and *ASXL1* exon 13 were screened with the use of the established high-resolution melting (HRM) assays and verified by Sanger sequencing as described before ^22–28^.

To verify whether two mutations found in the CLL-2423 sample are located in one or two different alleles, we sequenced individual colonies obtained from the sample. Briefly, *MIR142* was amplified by PCR with the following primers MIR142_F 5’-TAACTACAGCGGCCGCATCTCCGAAGCCCACAGTAC, MIR142_R 5’-TCCACTACGGAATTCCGGACAGACAGACAGTGCAG introducing the *Not*I and *Eco*RI restriction enzyme sites, respectively; PCR product was digested, gel purified, ligated into the pCDH-CMV-MCS-EF1-copGFP-T2A-Puro expression plasmid (CD513B-1, System Biosciences, Mountain View, CA, USA), and propagated in MAX Efficiency™ *E. coli* Competent Cells (Invitrogen, Carlsbad, CA, USA). Individual colonies (N = 10) after *MIR142* PCR amplification were sequenced using the Sanger method as described above. The sequencing revealed 6 clones with both identified mutations and 4 clones with the wild-type sequence.

## Results and discussion

**Figure 1:**
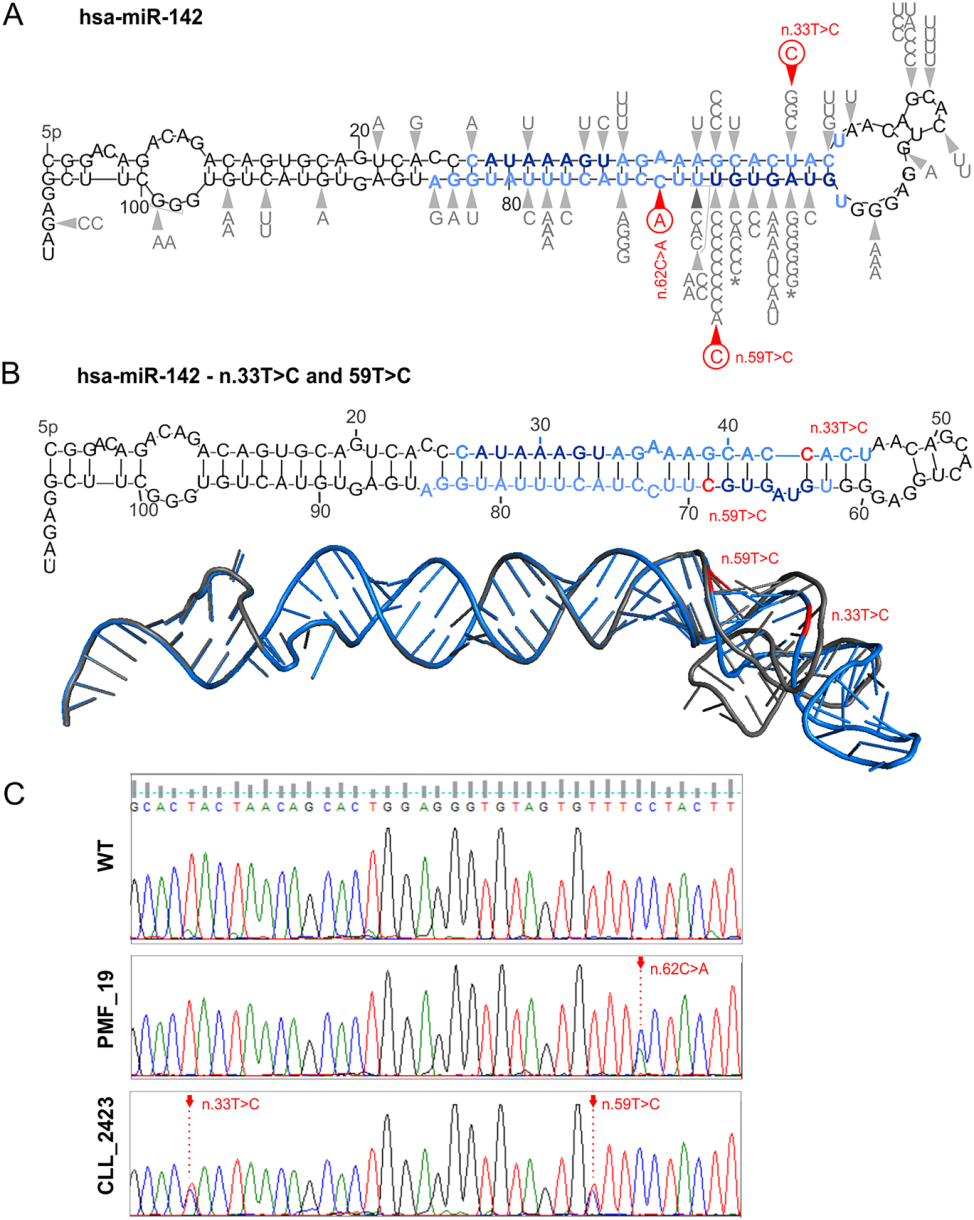
Characteristics of the *MIR142* mutations detected in this study. (**A**) Locations of the mutations on the secondary stem-loop structure (predicted with mfold^37^) of the wild-type miR-142 precursor. Blue font indicates miRNA duplex, dark blue indicates seed regions, and black indicates flanking regions and terminal loop. Gray symbols represent mutations identified before (based on^4^), and red-circled symbols indicate mutations identified in this study. (**B**) Secondary (above) and 3D (below) RNA structures adopted by the n.33T>C and n.59T>C mutant found in CLL. The 3D mutant structure (blue) is aligned with the corresponding wild-type structure (black). The mutations are indicated in red. The 3D structures were generated using RNAComposer^38^ and visualized in PyMOL (Schrödinger, LLC, New York, NY, USA). (**C**) Sanger sequencing results for wild-type and mutated samples.

To determine the occurrence of mutations in *MIR142* in MPNs, we analyzed a collection of 672 samples consisting of ET (n = 321), PV (n = 174), CML (n = 107), and PMF (n = 70). Additionally, we included CLL (n = 210) and AML (n = 47) samples, diseases for which the mutations in *MIR142* have been reported^4,29–32^. Screening was performed by Sanger sequencing of a PCR-amplified DNA fragment encompassing the *MIR142* gene (defined as described above). Altogether, we identified only 3 mutations: one mutation [chr17:58331257G>T[-]; n.62C>A] not reported before was identified in a PMF sample (PMF_19 patient; 1/70); two other mutations [chr17:58331286A>G[-], n.33T>C and chr17:58331260A>G[-], n.59T>C] were identified in one CLL sample (CLL_2423 patient; 1/210). The n.62C>A mutation is located in the miR-142-3p post-seed region in a symmetric one-nucleotide-bulge structure (Fig. 1A and C). Although the mutation does not induce any apparent change in the predicted secondary structure of the miRNA precursor, it may influence miRNA:target interactions as well as the stability of the miRNA itself^33,34^. Both mutations identified in the CLL sample are located in two established hotspots (n.33T>C, n.59T>C) and were previously described in CLL^29^. Both mutations are in the mature miRNA duplex, specifically in the seed region of miR-142-3p and in the post-seed region of miR-142-5p (Fig. 1A). Cloning of the PCR product and subsequent sequencing of individual clones revealed that both mutations are located in the same allele (6 out of 10 sequenced clones). TargetScan analysis predicted that the n.59T>C mutation severely affects miR-142-3p target recognition, disabling recognition of 55% (139/250) of its targets and creating 79 new targets. Whereas, analysis of the n.33T>C mutation showed that it substantially affects the structure of the miRNA precursor (Fig. 1B) and hence very likely affects processing of the precursor and miRNA release. All identified mutations have increased functional weight scores resulting from a location in crucial miRNA precursor elements (based on miRMut criteria defined before)^4,35,36^.

Briefly, the PMF_19 patient, a male aged 57 years old, was diagnosed with PMF grade 3 (MF3), with a bone marrow blast content of 3.5% and intermediate 2 risk of unfavorable disease outcome according to the Dynamic International Prognostic Scoring System. The patient had a normal karyotype, was negative for the BCR-ABL transcript, and was triple-negative for driver mutations (*CALR* exon 9, *JAK2* exon 12 and 14*,* and *MPL* exon 10), which is characteristic of up to 10% of PMF cases, usually with a less favorable prognosis^39^. Additionally, molecular analysis of *ASXL1* and *U2AF1* genes revealed c.1904_1927dup (p. Glu635_Gly642dup) in the former and c.470A>G (p.Gln157Arg) in the latter, which are considered unfavorable prognostic factors^40^. These additional, considered non-driver mutations were shown to contribute to disease progression, and ineffective hematopoiesis, and are taken into account in the genetically inspired prognostic scoring system (GIPSS) of PMF^41,42^. Co-occurrence of *ASXL1* and *U2AF1* mutations with mutations in *MIR142* has also been noted in AML^30^.

The CLL_2423 patient, a male aged 62 years old, was diagnosed with B cell CLL (IV stage according to Rai classification). No chromosome alteration was observed by nuclear FISH (including the chr17p/*TP53* gene). After 6 cycles of immunochemotherapy (Rituximab, cyclophosphamide, dexamethasone protocol), complete hematologic remission was achieved in this case. Of interest, two events of further disease relapse were noted, the latter with coexisting hyperleukocytosis and *TP53* deletion in 7% of nuclei tested.

As mentioned above, it was noticed before that *MIR142* mutations observed in AML coincide with and are functionally related to the R140Q hotspot mutation in *IDH2*^5,6^. However, in this study, we did not detect any *IDH1*/*IDH2* mutation, generally observed in 4% of PMF cases^43^, in any of the two samples (PMF_19, CLL_2423) with *MIR142* mutations.

Together, our results show that mutations in *MIR142* are generally very rare in MPN but that their frequency (~ 1.5%) in PMF may be in the range of that observed in CLL or AML ^4,29–32^. Nevertheless, further studies are needed to determine the real contribution of *MIR142* mutations to PMF. Despite the mostly negative nature of our results, this study complements knowledge about the occurrence of *MIR142* mutations in blood cancers and pancancer in general. Additionally, as functional studies of *MIR142* mutations were performed only in AML, in which the mutations are relatively rare, the confirmed recurrence of mutations in other hematologic cancers indicates that further studies are needed to fully understand the role of these mutations, especially in malignancies in which they are the most frequent, i.e., DLBCL and FL.

In summary, although we show that *MIR142* mutations may occur in MPNs, particularly in PMF, the frequency is very low (~ 1.5%). We also confirmed the occurrence of *MIR142* mutations in CLL, but similarly at a very low frequency.

## Data Availability

All data generated or analyzed during this study are included in this published article.
